# Assessment of the Food-Swallowing Process Using Bolus Visualisation and Manometry Simultaneously in a Device that Models Human Swallowing

**DOI:** 10.1007/s00455-019-09995-8

**Published:** 2019-03-06

**Authors:** Waqas M. Qazi, Olle Ekberg, Johan Wiklund, Reinhardt Kotze, Mats Stading

**Affiliations:** 1 Agrifood and Bioscience, Product Design and Perception, RISE, Research Institutes of Sweden AB, Göteborg, Sweden; 2 Department of Industrial and Material Sciences, Chalmers University of Technology, Göteborg, Sweden; 3 Diagnostic Centre of Imaging and Functional Medicine, Skåne University Hospital, Lund, Sweden; 4 Incipientus Ultrasound Flow Technologies AB, Frans Perssons Väg 6, 412 76 Göteborg, Sweden

**Keywords:** Deglutition and deglutition disorders, Shear rate, Rheology, Ultrasound velocimetry, Bolus manometry

## Abstract

The characteristics of the flows of boluses with different consistencies, i.e. different rheological properties, through the pharynx have not been fully elucidated. The results obtained using a novel in vitro device, the Gothenburg Throat, which allows simultaneous bolus flow visualisation and manometry assessments in the pharynx geometry, are presented, to explain the dependence of bolus flow on bolus consistency. Four different bolus consistencies of a commercial food thickener, 0.5, 1, 1.5 and 2 Pa s (at a shear rate of 50 s^−1^)—corresponding to a range from low honey-thick to pudding-thick consistencies on the National Dysphagia Diet (NDD) scale—were examined in the in vitro pharynx. The bolus velocities recorded in the simulator pharynx were in the range of 0.046–0.48 m/s, which is within the range reported in clinical studies. The corresponding wall shear rates associated with these velocities ranged from 13 s^−1^ (pudding consistency) to 209 s^−1^ (honey-thick consistency). The results of the in vitro manometry tests using different consistencies and bolus volumes were rather similar to those obtained in clinical studies. The in vitro device used in this study appears to be a valuable tool for pre-clinical analyses of thickened fluids. Furthermore, the results show that it is desirable to consider a broad range of shear rates when assessing the suitability of a certain consistency for swallowing.

## Introduction

Thickening of liquids for consumption is a common approach to manage and nourish individuals who are suffering from dysphagia [1, 2]. The thickeners used for dysphagia are based on the fundamental concept of increasing bolus consistency, thereby reducing the velocity of the flow during the swallowing process. This allows sufficient time for muscular adjustments in individuals who are suffering from dysphagia. Such individuals are susceptible to low-viscosity fluids [3]. In contrast, a bolus of too-high viscosity demands that extra force be exerted by the tongue and pharyngeal muscles to push the bolus through the oropharynx. Individuals who lack pharyngeal muscle or tongue strength may experience post-swallow residues [4], requiring a secondary clearing swallow [5]. Most of the published studies on pharyngeal bolus velocity report it as being in the range of 0.1–0.5 m/s at different locations in the pharynx. Higher velocities have been recorded for water, which decrease as the thickener concentration increases [6–8].

Clinical studies using ultrasound have demonstrated that increasing the bolus viscosity results in lower and flatter (i.e. less variation of the maximum and minimum velocities) velocity profiles above the epiglottis. The lower velocities are due to a high fluid viscosity, while the velocity profiles are flatter due to shear thinning [5]. Shear thinning, which is a term that is less familiar to the medical community, is crucial, as almost all fluid foodstuffs have viscosities that decrease with increasing shear rate (velocity) [1, 9].

Commercially available thickening powders are used to manage the delayed pharyngeal response. Thickening powders are usually gum based or starch based. Starch molecules swell upon hydration, thereby increasing the viscosity of a solution, whereas gums form a network of entanglements that arrest water. Starch-based thickeners have been shown to break down during digestion especially in oral phase due to amylase enzyme present in saliva [10], which results in decreased viscosity of the thickened fluid. Gum-based thickeners transit relatively unchanged during oral processing [11].

Thickeners, whether gum or starch based, are shear thinning, so the shear rate should always be mentioned for a given consistency of a fluid, as recognised by the European Society for Swallowing Disorders (ESSD) in its recently published White Paper [8].

Very little has been published on shear rate measurements for bolus transport in the pharynx. To our knowledge, the shear rate during swallowing has been determined in only a few simulation studies, e.g. those conducted by Meng et al. [12] and Salinas et al. [13]. Meng et al. used a 2D geometry for the simulation and only reported the shear rate at the UES. Simulation studies cannot capture the complexity of bolus flow in humans. Zhu et al. [7] studied commercial thickeners and glucose mixed with contrast media during video fluoroscopic analysis of three patients. From these analyses, the velocities and associated shear rates were calculated. The use of contrast media is restricted to clinical examinations, so the results might be different in practical situations when real fluid foodstuffs are swallowed.

To determine the bolus shear rate, we have applied a unique, non-invasive Ultrasound Velocity Profiling (UVP) technique [14]. UVP measures the real-time flow in tubes, ducts, and similar geometries. The technique is based on the reflection of ultrasound waves from reflecting particles/bubbles in a flowing fluid. Thus, UVP does not require any contrast media. UVP measures the velocity profile directly in a given geometry, and it can be applied to bolus flow to determine accurately the shear rate distribution during swallowing.

Manometry is an important tool for measuring pressure variations during swallowing using an in-dwelling catheter [15, 16]. Studies have shown that when the bolus volume and viscosity are increased, the recorded intra-luminal pressure also increases, which means that greater force is needed to transport a bolus of larger volume [17, 18]. Ergun et al. studied the UES shape, velocity, and bolus volume interactions using ultra-fast computer tomography, and they concluded that 15 ml of compulsory air were swallowed with the bolus [19]. Bolus consistency influences the maximum pharyngeal pressure, UES contraction, UES opening/closing duration, and the duration of pharyngeal pressure [20].

The well-known catheter method of pressure measurement is invasive, and under certain conditions it might obstruct the bolus flow [21, 22]. Moreover, with the catheter method, laborious calibration steps must be followed for individual patients [18]. Furthermore, ethical concerns regarding safety always arise in clinical studies [23]. Consequently, improved thickener formulations and the incorporation of novel rheological attributes, such as fluid elasticity, yield stress, and shear thinning (as proposed in the ESSD White Paper), are challenging to test on patients directly. As mentioned before, clinical studies can produce different results even when studying the same hypothesis. Variations in the results are mainly attributed to the complexity of the swallowing process and inter-subject variability. In a previous study, we examined the influence of elasticity on safe swallowing for patients with dysphagia [24]. Fluid elasticity promoted safe swallowing, although the inter-subject variability was large. Therefore, an in vitro swallowing device that can generate a realistic bolus flow and that allows the performance of clinical types of measurements represents a perfect balance between the two extremes of in vitro simulations and medical examinations.

This study describes a unique approach to performing thorough investigations of in vitro swallowing using manometry and bolus visualisation techniques simultaneously and non-invasively.

## Materials

A powdered thickener from Nutricia Nordic AB (Stockholm, Sweden) was used in the experiment. Four different bolus consistencies (Table 1) of the given thickener were used for UVP, while two of them were used for the manometry. The thickener was added to water so that the consistency was set in the range of 0.5–2.0 Pa s at a shear rate of 50 s^−1^, thereby covering the consistency range from lower honey thick (51–350 mPa s) to pudding thick (> 1750 mPa s), according to the National Dysphagia Diet (NDD) scale [25]. Since the three viscosities used in the current experiments lie in the honey-thick range (351–1750 mPa s), this range is further categorised into low, medium, and high consistencies (Table 1), to simplify the interpretation in the *Discussion*.

**Table 1:** Amounts of thickener powder added to 100 ml water to achieve the different viscosities

Viscosity at 50 s^−1^ (Pa s)	Added powder (g)	NDD description
0.5	4.9	Low, honey thick
1.0	5.8	Medium, honey thick
1.5	7.1	High, honey thick
2.0	7.8	Pudding thick

The viscosity was measured using a conventional ARES-G2 instrument from TA Instruments (New Castle, DE, USA). A cone and plate geometry was used with diameter of 40 mm and angle of 0.04 radians. The shear rate was varied from 1 s^−1^ to 1000 s^−1^. The temperature was set to 25 °C. The consistencies of the liquids were all shear thinning, and when fitting the power law model *n* = 0.33. The power law model describes shear stress = constant × rate^flowindex^. Therefore, in subsequent sections, the mentioned viscosity is always at a shear rate of 50 s^−1^.

## Methods

**Fig. 1:**
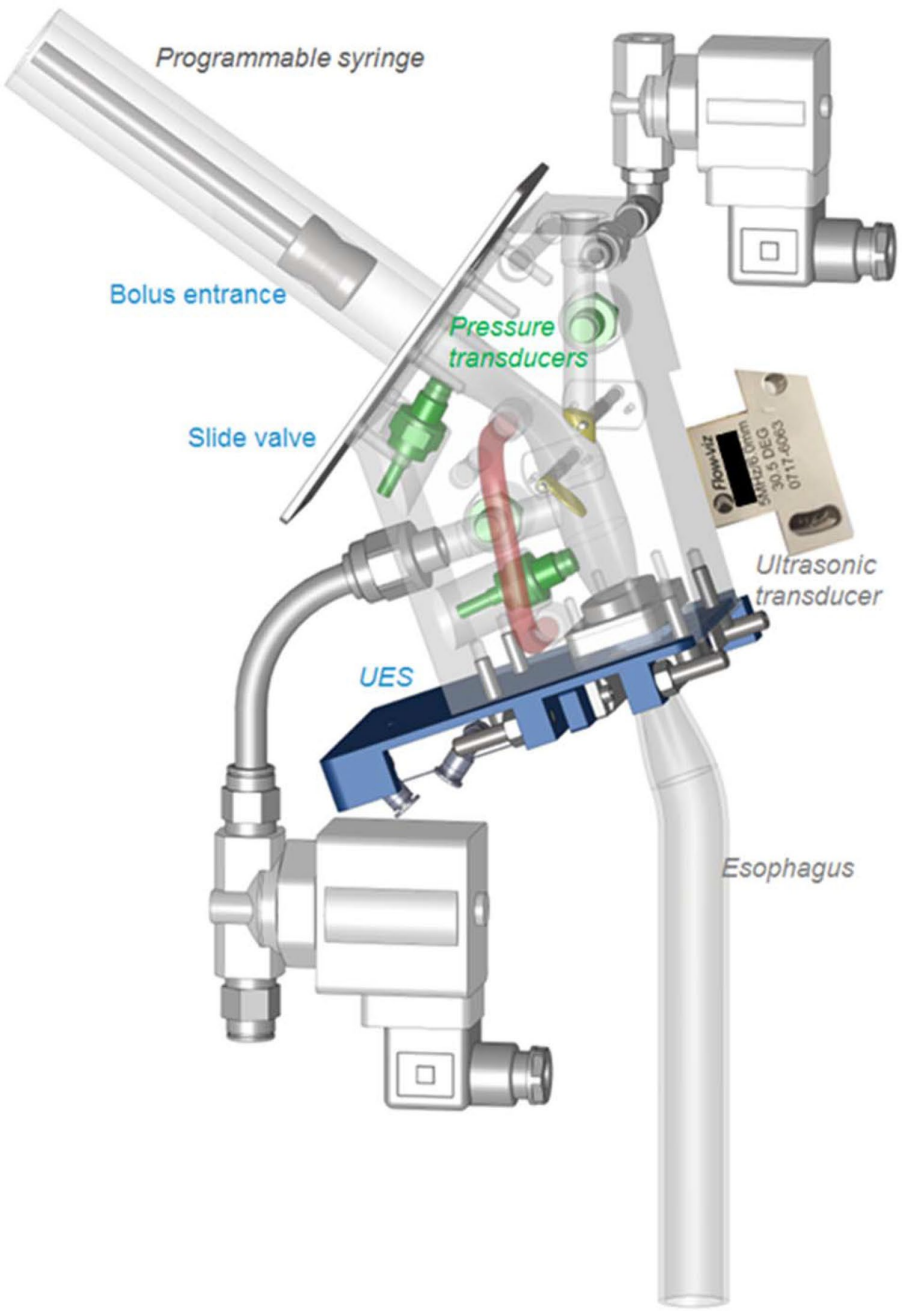
Sketch of the model pharynx showing the geometry, different valves temperature regulation (red-tube), ultrasound, and pressure transducers installed

The in vitro simulator, called *the Gothenburg Throat,* is described in detail in a separate paper [26]. Nevertheless, to guide the reader, a brief account of how the simulator works is provided here (see Fig. 1).

The given fluid is stored in a tank that is connected to the simulator. The fluid is transported to a syringe pump, simulating the oral phase. A bolus of set volume and velocity is injected into the model pharynx, mimicking the thrusting action of the tongue. While filling the syringe, the slide valve is kept closed to prevent fluid flow by gravity until the barrel is filled with the fluid. The slide valve is opened momentarily, ensuring that the fluid flows only due to the thrust exerted by the syringe. Two valves mimic the opening to the larynx and the nasopharynx, a movable epiglottis closes during swallowing, and a clamping valve mimics the upper oesophageal sphincter (UES). The UES is in the closed position from the start, and is opened for the model bolus to eject while the epiglottis from its original open position is closed. The epiglottis does not hermetically seal the entrance to the larynx opening. As the bolus passes the pharynx, the velocity profile is measured by UVP, while the pressure transducers record the pressures at four locations. A 3-s interval is set between each bolus injection.

### In Vitro Device Settings

In the current work, the bolus volume was 15.0 ± 2.5 ml, unless otherwise stated. A camera (DSC-RX100M5; Sony, Tokyo, Japan) installed at a distance of 10 cm from the flow simulator was used to capture the syringe speed, which was equal to the initial bolus speed. In this work, the piston speed, as regulated from the compressed air regulator, was set to remain at 1.25 ± 0.03 m/s, irrespective of the bolus volume and viscosity. During photographic sequencing of the bolus flow, the camera was operated in the high-frame-rate mode, capturing images at 50 frames/s. The bolus was dyed blue for better contrast, ensuring that colour addition did not influence the bolus rheology. The acquired slow-motion videos were analysed using Media Player, MPC-HC ver. 1.7.13 in high-precision mode. With knowledge of the frame rate and distance travelled by the bolus head, the velocity can be calculated.

## Shear Rate Calculation

The velocity profiles were acquired using the latest UVP instrument from Incipientus Ultrasound Flow Technologies AB (Gothenburg, Sweden). A novel non-invasive transducer was specifically designed for the *Gothenburg Throat Simulator*, which consists of a 5-MHz, 6-mm piezo transducer that generates a 30.5° beam in the pharynx.

The UVP transducer captured velocity profiles in real time. From the gradient of the velocity profile, *v*(*r*), the shear rate ($\dot{\gamma }$) was calculated as a function of the radius [14, 27–29]:

2 $$\dot{\gamma } = - \frac{{{\text{d}}v(r)}}{{{\text{d}}r}}.$$

**Fig. 2:**
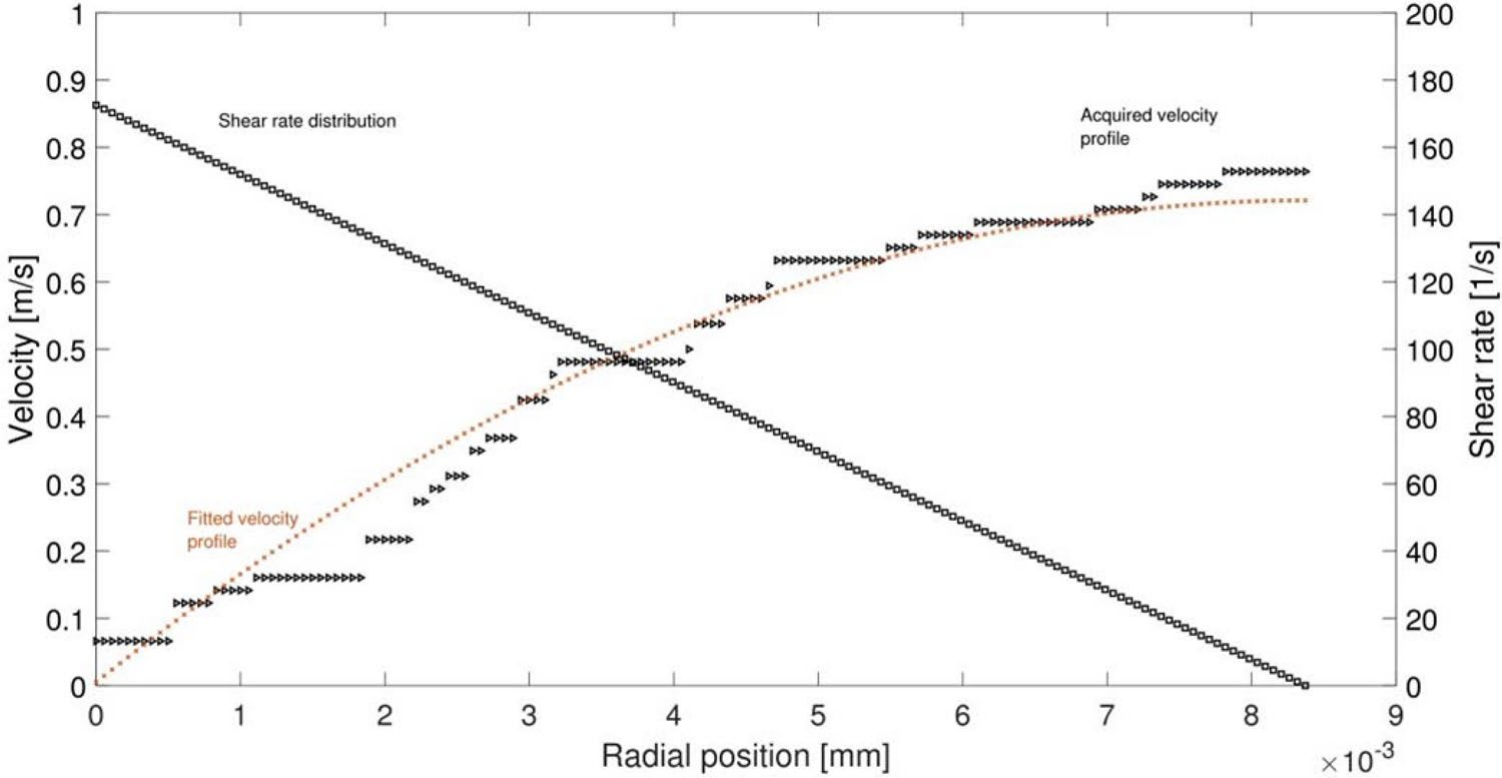
Method used for shear rate estimation. The *x*-axis shows the radial position in the tube from the wall to the centre, while the *y*-axis (left) shows the acquired Doppler-shifted velocity profile, to which a polynomial is fitted. Th *y*-axis (right) shows the shear rate distribution in the bolus

To determine the gradient and smoothen the experimental data, a second-order polynomial was applied on the experimental data. Figure 2 shows an example of the shear rate calculation from the experimental data. The shear rate distribution inside the bolus is shown, and the maximum shear rate reported here is calculated from the velocity gradient at the wall of the model pharynx.

**Fig. 3:**
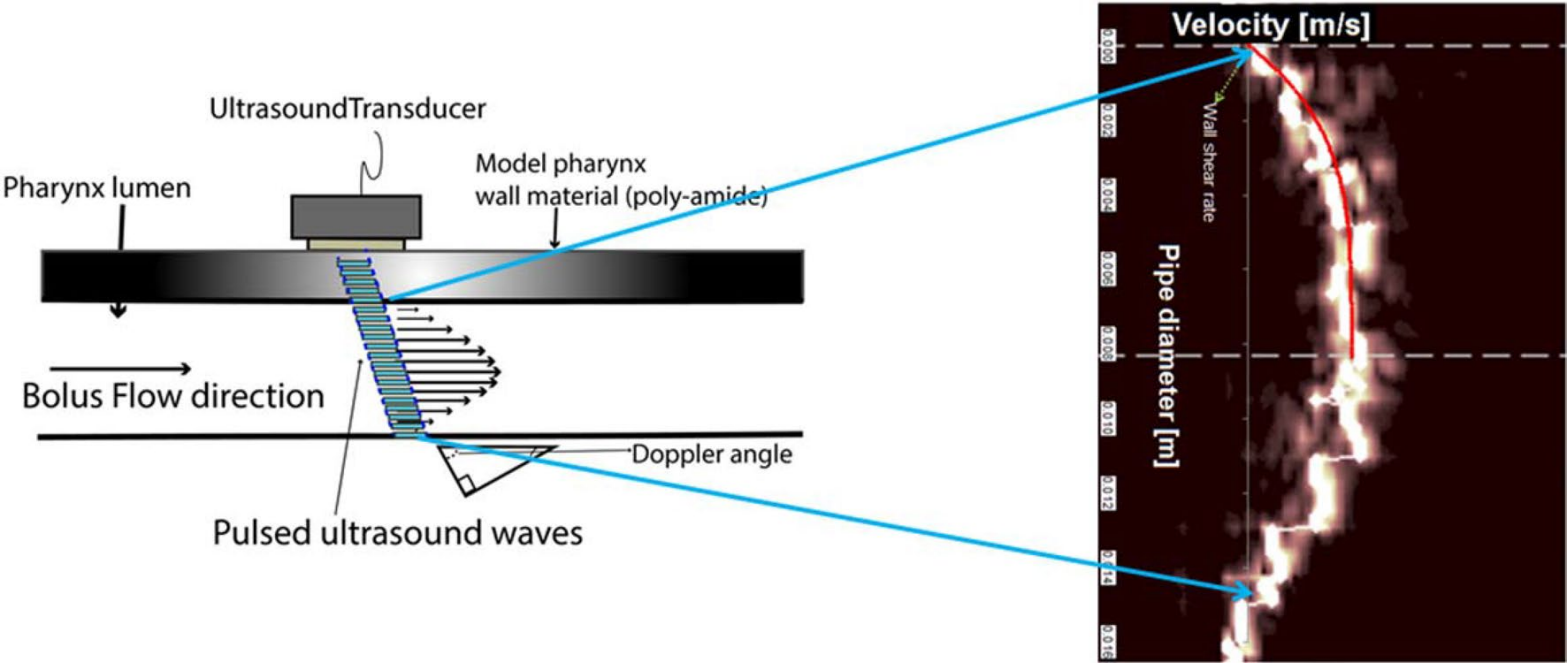
Schematic showing bolus flow inside the model pharynx, with the Doppler line created by the ultrasound transducer, giving an actual velocity profile and the location of the wall position where the maximum shear rate is measured

Only half of the velocity profile closest to the transducer is needed to calculate the shear rate distribution, as the geometry is assumed to be symmetrical with the other half of the pipe. The data acquired closer to the transducer side are more accurate, since the ultrasound energy reduces with distance in the other half, due to absorption and attenuation. Figure 3 is presented as an example of data acquisition with the ultrasound transducer used in the present work.

The base frequency of the ultrasound transducer used in current work was 5 MHz. On the average, 128 ultrasound pulses were used to construct each velocity profile. Based on off-line experiments, the sound velocity, radius at the short axis of the elliptical geometry, and the Doppler angle were set at 1500 m/s, 8.4 mm, and 69°, respectively.

### Manometry Analysis

Manometry was performed using the pressure transducers and monitoring software (Oscilloscope from Pico Technology, Cambridgeshire, England). Pressure transducers at three locations (pharynx entrance, mid-pharynx, and close to the UES) inside the modal pharynx and the one at nasal cavity were calibrated against a digital reference pressure transducer for air using the DPI 705 unit (Amtele Engineering AB, Stockholm Sweden). The accuracy of the transducers was < 6% in the pressure range of 1–48 kPa, which was considered acceptable for this application. The pressure transducer in the nasal cavity does not come in contact with the bolus and is used as a control to differentiate bolus pressure from air pressure. Thus, corrected pressure values are reported here. Pressure peak duration (seconds) was measured by calculating the onset and offset of pressure wave generation.

### Statistical Analysis

Comparison of mean values was performed using the *t* test in the Microsoft Excel 2010 software.

## Results

### Velocity Profiles and Shear Rates During Bolus Flow

**Fig. 4:**
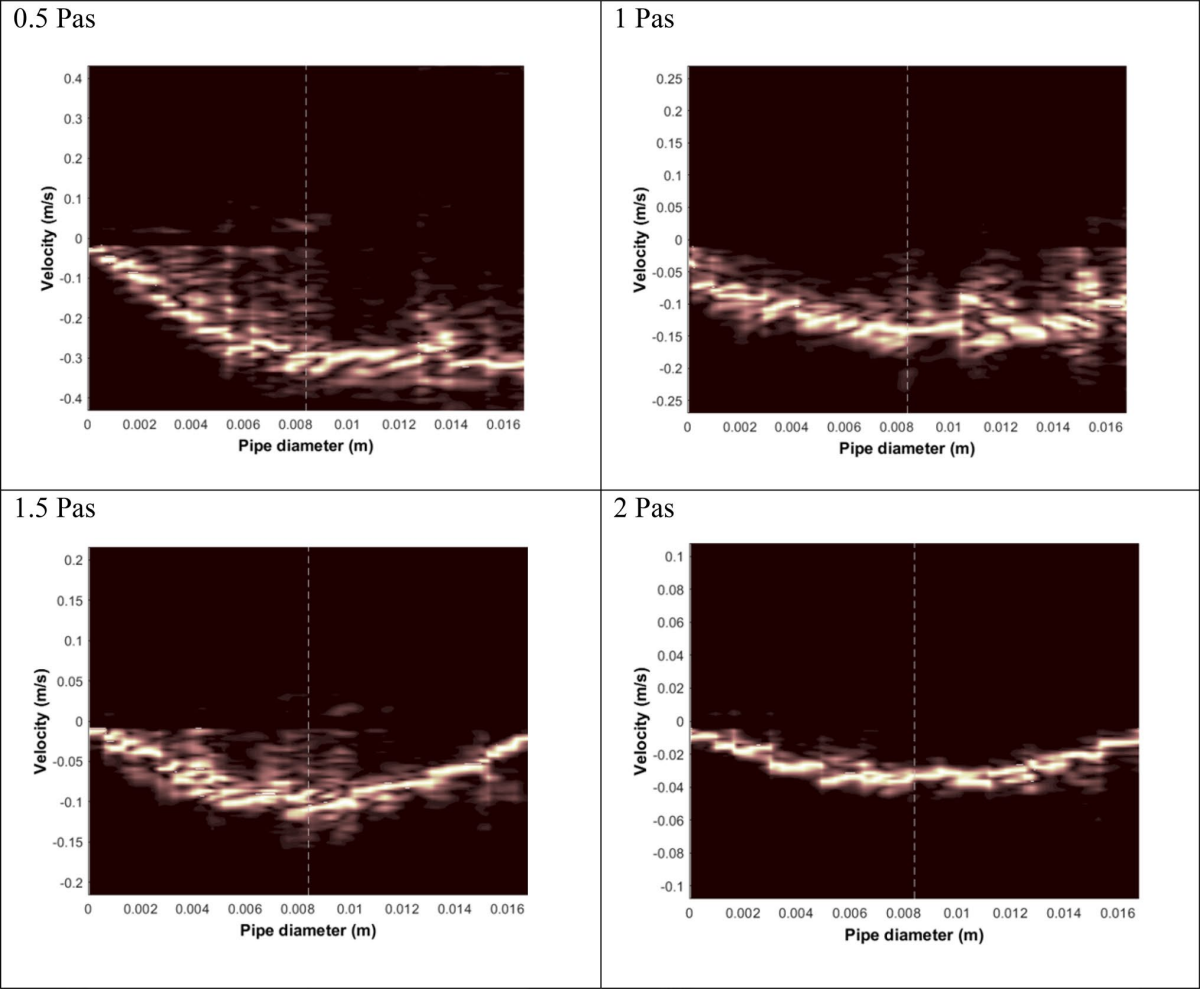
Velocity profiles acquired with ultrasound velocimetry using the Nutilis thickener at viscosities of 0.5, 1.0, 1.5, and 2.0 Pa s at a shear rate of 50 s^−1^

The velocity profiles of the boluses thickened to consistencies of 0.5, 1.0, 1.5, and 2.0 Pa s (at 50 s^−1^) are shown in Fig. 4 as power spectra, where the brightness level is proportional to the energy of the ultrasound beam. The velocity profiles were measured from the transducer side to the centre of the model pharynx. Negative velocities were noted when the bolus flow took place in the direction opposite to the ultrasound beam, which is inclined at 69°. The average fluid velocities ranged from 0.046 ± 0.02 m/s to 0.48 ± 0.05 m/s, increasing in the order of decreasing bolus consistency (Table 2). The differences in velocities between the boluses with consistencies in the ranges of 0.5–1.5 Pa s and 0.5–2.0 Pas were statistically significant (p=0.05). The velocity profiles acquired for low-viscosity fluids are somewhat noisy, as bolus transport is slightly different than, for example, a fluid that is flowing continuously. Bolus transport is a rapid process, occurring in less than 1 s in healthy subjects and in the in vitro simulator. To capture this rapid movement, faster data acquisition and faster on-screen display are desirable. Therefore, a lower number of ultrasound pulses (128) were used to capture a single Doppler spectrum, i.e. velocity profile (displayed in Fig. 4). Furthermore, air accumulated inevitably during the pumping of boluses that caused a decrease in the Doppler frequency range due to the slower velocities of the entrained bubbles. It is noteworthy that most of the Doppler noise was in the lower velocity range, especially in the cases of boluses with low viscosities (0.5 Pa s and 1.0 Pa s). The noise levels decreased for high-viscosity boluses, such as those with a consistency of 2.0 Pa s, due to the high number of particles and the presence of less air in the more-viscous boluses.

**Table 2:** In vitro shear rates and velocities at different viscosities of a fluid

Viscosity at 50 s^−1^	Maximum velocity (m/s)	Shear rate (s^−1^)
0.5	0.482 ± 0.049^a^	208 ± 45.8^a^
1.0	0.208 ± 0.094^ab^	73.8 ± 20.3^b^
1.5	0.096 ± 0.043^bc^	23.6 ± 4.12^bc^
2.0	0.046 ± 0.009^bd^	13.3 ± 4.36^d^

Values shown are mean  ±  standard deviation of minimum five repetitions. Different letters within the same column shows statistically significant difference (*p *< 0.05)

In general, the increased velocity that resulted from a low viscosity yielded higher shear rates, as expected. A significantly higher (*p* = 0.05) shear rate (~ 209 s^−1^), calculated from the gradient of the velocities recorded at the wall, was seen for the bolus with the lowest consistency. The pudding consistency (viscosity of 2.0 Pa s at 50 s^−1^), which was the highest consistency used in the current work, yielded the lowest shear rate of 13 ± 6 s^−1^. In addition, the difference in shear rate was statistically significant (*p* = 0.05), as compared to the other consistencies. The high syrup consistency (1.5 Pa s at 50 s^−1^) yielded a wall shear rate of ~ 24 s^−1^, while the medium honey-thick consistency gave a wall shear rate of ~ 74 s^−1^; these shear rate values are statistically significant (*p *= 0.05) than those for the least and highest bolus consistencies, 0.5 Pa s and 2.0 Pa s, respectively, used in the current work.

#### Optical Bolus Visualisation

**Fig. 5:**
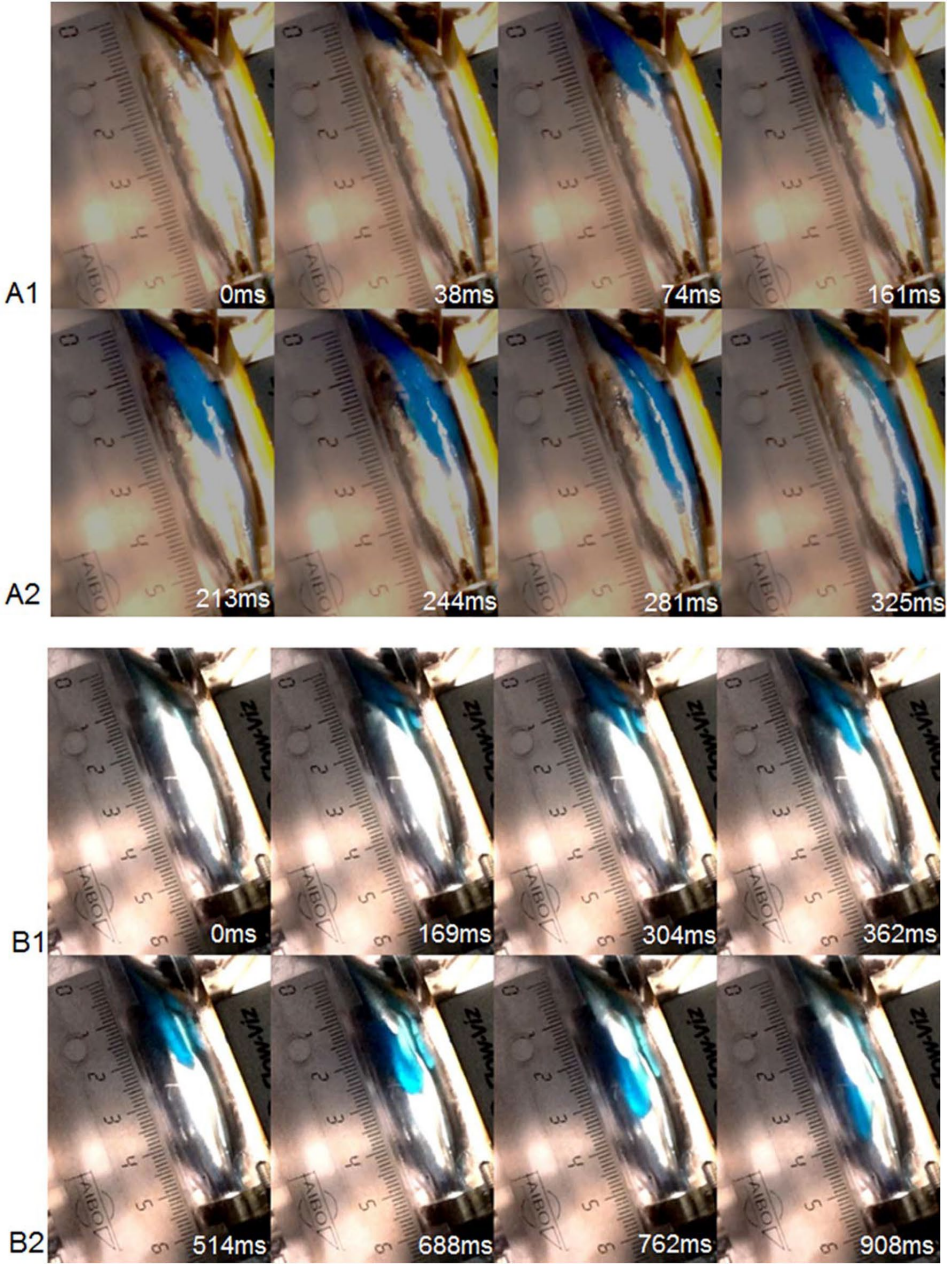
Photographic image sequences of bolus ejection with time for boluses with viscosities of 1.0 Pa s (**A1**, **A2**) and 2.0 Pa s (**B1**, **B2**), having a shear rate of 50 s^−1^

Figure 5 shows the photographic image sequences for the model bolus flows for the fluids with two different consistencies that were injected into the model pharynx. The model pharynx is empty at *t *= 0 ms. The bolus gradually enters the pharynx and flows towards the UES with time. Low-viscosity fluids (Fig. 5, A1 and A2) of 1.0 Pa s showed a transit time of 325 ms. In contrast, the bolus with pudding-like consistency (Fig. 5, B1 and B2) with a viscosity of 2.0 Pa s, demonstrated slower movement, resulting in delayed pharyngeal exit, i.e. a transit time of about 1 s.

## Manometry Results

### Influences of Fluid Viscosity and Syringe Speed on Pressure Levels in the Model Pharynx

**Fig. 6:**
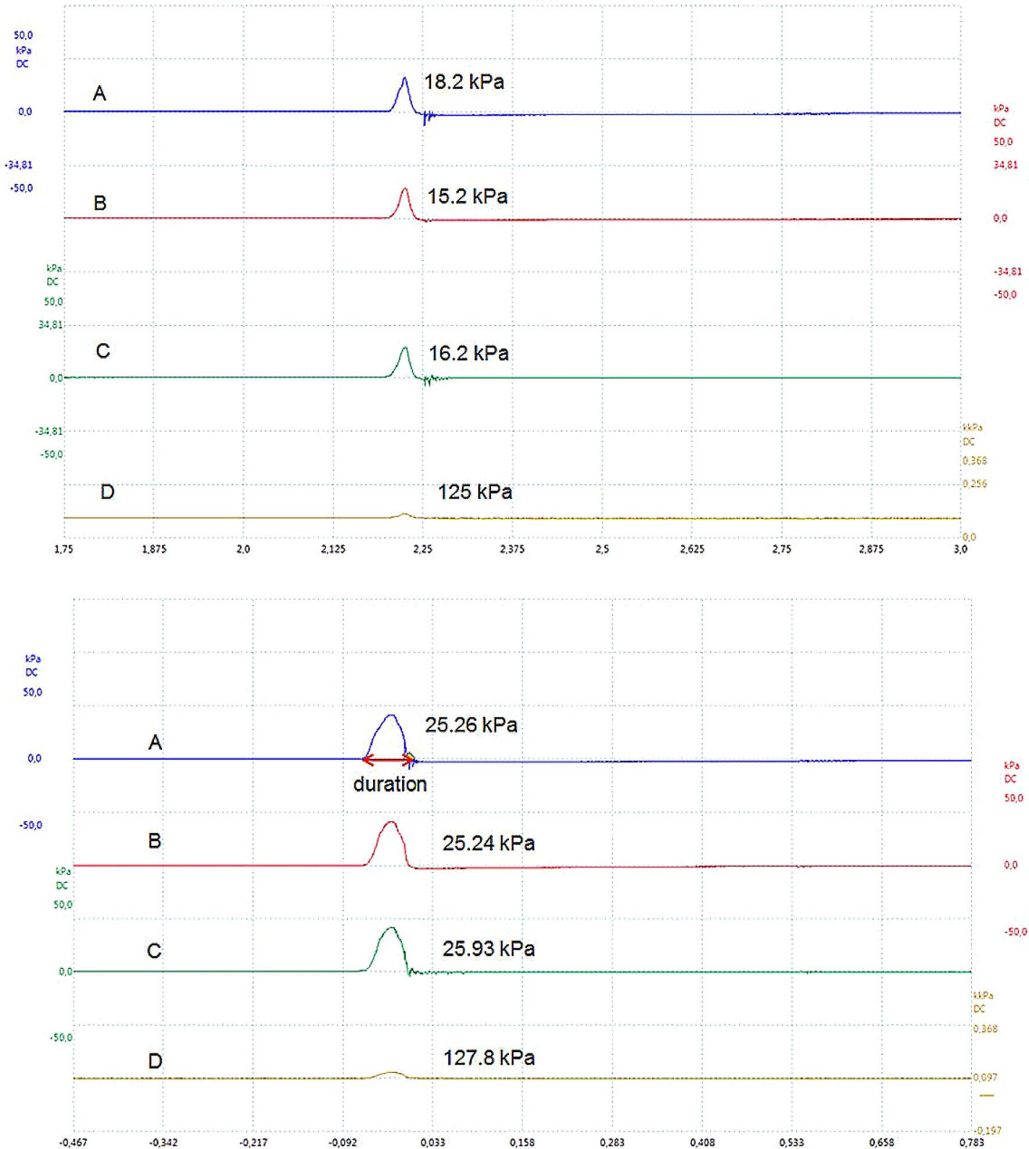
Typical graph recordings for the manometry results when a variable velocity was applied. On the *x*-axis is the time (seconds) and on the *y*-axis is pressure (kPa). A negative time is the time recorded prior to the trigger. The upper panel shows the waveform of a 1.0-Pa s bolus, while the lower panel shows the 2.0 Pa s wave-forms. Pressure wave duration is indicated with a red arrow. The different letters in the graphs indicate the maximum pressures recorded at the following locations in the model device: A, pharynx entrance; B, mid-pharynx; C, just above the UES; D, sensor located at the nasal cavity, thus showing the overall pressure due to air plus bolus flow

Table 3 shows that the high-viscosity fluid (2.0 Pa s at 50 s^−1^) resulted in higher intra-bolus pressures at all locations in the model pharynx. The pressure values were always slightly higher (statistically insignificant at p=0.05) at the pharyngeal entrance (Fig. 6, A and B). The mid-pharynx transducer was mounted behind the epiglottis, which was closed and thereby restricted the air flow. The short pressure pulses, therefore, do not allow sufficient time for equilibration, resulting in slightly lower pressure readings.

**Table 3:** Pressures applied (kPa) with respect to the different fluid viscosities at different locations in the in vitro swallowing model

Viscosity of fluid at 50 s^−1^	Pharynx entrance (kPa)	Mid-pharynx (kPa)	Near UES (kPa)
1.0 Pa s	20.19 ± 6.42^a^	18.60 ± 6.41^a^	19.07 ± 6.67^a^
2.0 Pa s	26.10 ± 3.93^b^	23.52 ± 4.96^b^	25.53 ± 4.20^b^

Results shown are mean and standard deviation (±) of a minimum of five measured pressure values. Different letters within the same column indicate statistically significant differences (*p *< 0.05)

To demonstrate the actual data acquired from the measuring system and the shapes of the pressure peaks, Fig. 6 is presented as a representative example. The more-viscous bolus resulted in visibly higher intra-bolus pressures at the different locations for the high-viscosity fluid, and the opposite phenomenon was seen for the low-viscosity fluid. A detailed explanation for this observation is provided in Table 3. The two panels of Fig. 6 (upper and lower panels) further show that the overall pressure increases (location D) above the atmospheric pressure (~ 100 kPa) inside the model pharynx as a result of the bolus flow. Furthermore, Fig. 6 shows a clear difference in the pressure peak width; the pressure peak is broader for the fluids with consistency of 2.0 Pa s, when presented on the same time scale.

### Effect of Bolus Volume

**Fig. 7:**
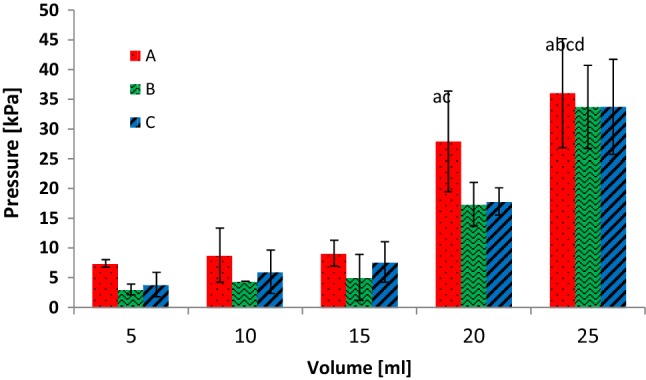
Mean and standard deviation of the pressure recorded as a function of bolus volume at different locations inside the model pharynx: *A* pharynx entrance, *B* mid-pharynx; and *C* close to the UES. Only the significantly different results with respect to volume at Transducer A are lettered. ^a^*p *< 0.05 compared to the 5-ml bolus; ^b^*p *< 0.05 compared to the 10-ml bolus; ^c^*p *< 0.05 compared to the 15-ml bolus; ^d^*p *< 0.05 compared to the 20-ml bolus

Bolus volume had a profound effect on the pressures recorded in the model pharynx (Fig. 7). The data could be categorised into two sets: low and high bolus volumes, i.e. 5, 10 and 15 ml are considered as low-volume boluses, while 20 ml and 25 ml are regarded as high-volume boluses. In the low-bolus-volume category, the pressure values increased incrementally but not significantly (*p *< 0.05; Transducer A). Within the high-bolus-volume category, the pressure values were significantly different (*p *< 0.05; Transducer A) between the 20-ml and 25-ml volumes. Between the high- and low-category boluses, statistically significant (*p *< 0.05; Transducer A) differences were noted for all the volumes, except between the 10-ml and 20-ml volumes. Similar trends of high volume and high pressure were noted for the transducers located at the mid-pharynx and pharynx exit. The pressure values were lower at the mid-pharynx with bolus volumes of 5, 10, 15, and 20 ml, due to the location of the transducer. The mid-pharynx transducer is located behind the epiglottis. The epiglottis was closed to prevent fluid flow into the model airways, thereby restricting the air flow and resulting in a lower measured pressure. The pressure levels at the UES were higher than at the mid-pharynx and lower than at the model pharynx entrance with respect to the different fluid volumes.

### Effect of Pressure Peak Duration at the UES Due to a Change in Viscosity

The pressure peak duration was longer for an increased fluid viscosity, as presented in Table 4. This effect was significant (*p *< 0.05) at the pharyngeal entrance and exit sensors of the model pharynx. At the mid-pharynx, the pressure peak duration for the 2-Pa s fluid was longer than that for the 1-Pa s fluid; however, this difference was not statistically significant (*p *< 0.05).

**Table 4:** Mean pressure duration (msec) across the transducers at different fluid viscosities

Viscosity of fluid at 50 s^−1^	Pharynx entrance (ms)	Mid-pharynx (ms)	Near the UES (ms)
1 Pa s	41.12 ± 14.73^a^	38.54 ± 14.55^a^	36.52 ± 11.01^a^
2 Pa s	61.53 ± 2.56^b^	55.78 ± 5.59^a^	59.94 ± 4.76^b^

Results shown are mean and standard deviation (±) of a minimum of five values. Different letters within same column indicate statistically significant differences (*p *< 0.05)

### Influence of UES Area Contraction

**Fig. 8:**
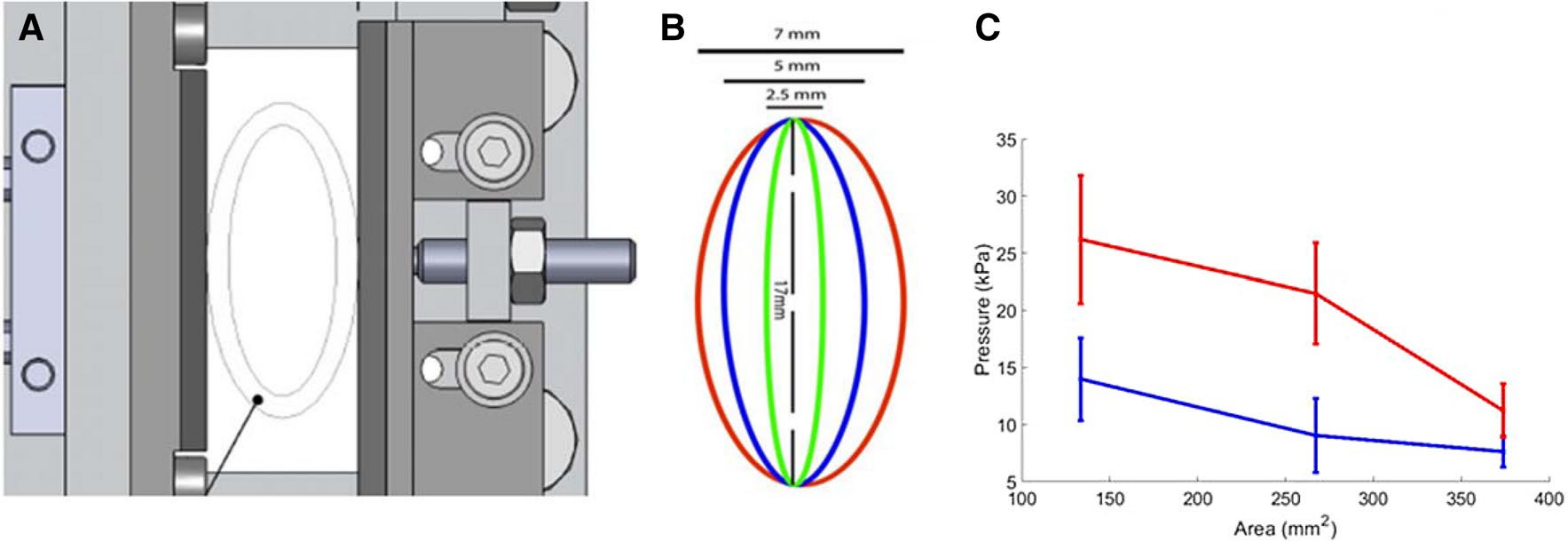
Changes in UES area and influences on pressure applied at the UES. **a** Original drawing of the model UES. **b** Area modification. **c** Changes in pressure with respect to consistencies of 2 Pa s (red line) and 1 Pa s (blue line) at 50 s^−1^ at the lower-most pressure transducer in the model pharynx

The elliptical area of the UES (Fig. 8, A and B), which originally was ~ 374 mm^2^, was decreased to 267 mm^2^ and 133 mm^2^ to simulate UES area contraction and the subsequent influence of pressure in the region, as shown in Fig. 8C. Two levels of fluid consistency, 1 Pa s and 2 Pa s, were used to study this effect at the lower-most pressure transducer in the model pharynx. As expected, decreasing the area of the UES resulted in increased pressure build-up at the UES. This effect was more pronounced for the fluid with viscosity of 2 Pa s.

## Discussion

This work involved a thorough investigation with an in vitro type of swallowing analysis that takes both bolus visualisation and manometry into account. The acquired bolus head velocities (0.48 ± 0.05 m/s) noted here are similar to those observed in clinical studies, such as those conducted by Tashiro et al. [3] (0.246–0.488 m/s) and Hasegawa et al. [30] (average velocity, 0.46 m/s). Both of these previous studies used the Pulsed Ultrasound Doppler technique, as was the case in the current study. Typical values from studies of bolus velocities in the pharynx lie in the range of 0.1–0.5 m/s [7]. In the present study, the largest reduction in velocity was found for the bolus with pudding consistency (2 Pa s). A similar observation was made by Rofes et al. [31] for a xanthan gum-based thickener, the same one as used here. In the present work, the ultrasound beam was directed towards the bolus flow above the epiglottis, i.e. in the meso-pharyngeal region. The velocity profile and subsequent shear rate are most interesting in the meso-pharynx due to the higher risk of aspiration in individuals who have slower airways closure. The thickened fluids used in dysphagia management are always shear thinning, i.e. the bolus will transit faster and be perceived to be thinner. Shear rates of > 200 s^−1^ for low honey consistency-thick viscosity and ~ 75 s^−1^ for medium honey-thick viscosity are above the current level of 50 s^−1^ mentioned in the NDD scale. Thickening of fluids to highly honey-thick and pudding-thick consistencies (viscosities of 1.5 Pa s and 2 Pa s, respectively, at 50 s^−1^) resulted in shear rates of < 50 s^−1^. The therapeutic effects of thickeners are linked to slowing of the bolus velocity through the pharynx [31]. This is further explained in our work by the low shear rate, which increases the perception of thickness with high honey-thick and pudding consistencies.

The bolus flow is dominated by the viscous forces, evidenced as low Reynolds number. The Reynolds number, $\left( {\text{Re} = \frac{\rho \cdot D \cdot v}{\mu }} \right)$, where (*ρ*) is the measured density; (*v*) is the velocity; and *D* is the hydraulic diameter, indicates whether a flow is dominated by viscous or inertial forces. It was calculated as ~ 20 for the least-viscous bolus having a viscosity of 0.5 Pa s at 50 s^−1^, with (*ρ*) = 1014 kg/m^3^, *v*=0.48 m/s (from ultrasonic analyses), and *D *= ~ 0.02 m (taking into account the non-circular geometry of the pharynx). A higher Reynolds number would reflect greater fluctuations in the velocities. The velocity profiles presented in Fig. 3 are clearly more stable, especially those acquired with high-consistency boluses, indicating that the estimated Reynolds number is reasonable. Thus, the thickened fluids used for dysphagia management provide a sufficient increase in viscosity to ensure that the bolus flow is dominated by viscous forces. This finding is predicted and discussed in detail by Burbidge et al. [5].

Bolus consistency, which is a manifestation of internal resistance to flow, is the best-known strategy for managing dysphagia. The main influence noticed in clinical trials is the longer bolus transit time, or in other words, slower resultant velocities with high-consistency boluses, as observed in our UVP measurements.

Photographic sequencing of bolus transport was performed to follow and confirm the physical events that occur inside the model pharynx, including the simultaneous detection of the pressure peak as the bolus hits the transducer and the disintegration of boluses having different compositions. When the photographic image sequences were analysed for transit times, they were found to be in good agreement with the velocity profiles determined using the UVP technique. The transit times measured from the photographic image sequences were 325 ms and 908 ms for the boluses with consistencies of 1 Pa s and 2 Pa s, respectively. Knowing the length of the model pharynx and the velocities form the UVP technique; the transit times are calculated as approximately ~250 s and ~1 s, respectively, for the two displayed consistencies of 1 Pa s and 2 Pa s. Clinical studies have reported typical pharyngeal transit times as short as 100 ms [32] and as long as 1 s as the bolus consistency increases. Therefore, the transit times reported here are within the range reported in clinical studies.

Adaption to bolus consistency was simulated in our experiments by keeping the syringe speed the same which is regulatable in the *invitro* device. This resulted in an increased intra-bolus pressure with increasing bolus consistency, as expected from the fluid mechanics. However, from the biological perspective, this is not always the case, as multiple factors, such as peristaltic action, arrival of the contraction wave, laryngeal movement, and epiglottal and UES movements, are involved. This explains why, when the body acts like a machine, e.g. the tongue thrust pushes the bolus towards the pharynx; higher pressures in the pharynx entrance are expected. These in vitro manometry results are not directly comparable to the results of the in vivo examinations. The swallowing process in humans is obviously much more complex than that in the device we used for this work. For this very reason, clinical studies do not always produce reproducible results with respect to changes in bolus consistency. For instance, in the clinical study conducted by Butler et al. [20], no significant association was detected between bolus consistency and the pressure values. Contrarily, Lan et al. [22] reported results opposite to those found in the current study, i.e. that a water bolus applies more pressure than do thick liquids. The explanation given by Lan et al. [20] was that the swallowing muscles act as a buffer to control the free flow of water. To accomplish this, the contractile muscles have to contract more to control the free flow of water, which results in a higher applied pressure.

The change in pressure is not proportional to the change in viscosity due to the fluid being shear thinning (*n* = 0.33). The tendency towards increased intra-bolus pressure with increasing bolus consistency was the same at every location in the model pharynx. The higher intra-bolus pressure at the entrance transducer demonstrates that the flow is pressure-driven and not gravity-induced in the simulator.

Bolus volume is an important variable that has been considered in many studies [18, 33–36]. A low bolus volume may trigger uncontrolled swallowing, whereas a high bolus volume for patients with neurological problems increases the risk of penetration/aspiration [34]. Hoffman et al. [36] noticed an increase in the velopharynx pressure when the bolus volume was increased from 5 ml to 20 ml. However, using a bolus volume of up to 10 ml, Butler et al. [18] did not observe any significant differences in pharyngeal pressure. The bolus volume was varied between 3 ml and 10 ml, as opposed to between 5 ml and 25 ml here. We speculate that the bolus volumes of up to 15 ml used in most clinical studies do not have significant impacts on the pharyngeal pressure and the subsequent biomechanical events, such as UES opening duration and total swallow duration, while bolus volumes > 15 ml do influence these events.

A longer pressure duration for a viscous bolus was observed by Al-Toubi et al. [16], analogous to the findings of the present study. In the present study, higher values for pressure duration were noted, as compared to those reported by Al-Toubi et al. [16]. This is due to the much higher viscosities of the boluses (1 Pa s and 2 Pa s), as compared to the swallowing of saliva and water investigated by Al-Toubi and colleagues. A similar study performed by Lin et al. did not notice any significant differences in pressure duration associated with bolus consistency or volume [34].

According to Bhatia and Shah [37], ~ 70% of patients with dysphagia have a malfunctioning UES, and the intra-bolus pressure is influenced by the consistency and area of the bolus. In the present work, decreasing the UES area and increasing bolus consistency yielded results similar to those reported by Chen et al [21]. Those authors described how the pressure values at the UES contraction increased twofold as the consistency increased from water to solid boluses. A similar trend was noticed in the current study, where a nearly twofold increase in intra-bolus pressure was noticed at all three levels of areas modified. The pressure values reported here are measured slightly above the UES, at the beginning of the UES contraction, and the sensor is embedded in the model pharynx wall, as opposed to the situation in clinical studies where a manometry catheter is used.

Our results suggest that by creating a realistic bolus flow in a pharyngeal geometry similar to the biological counter-part, concrete conclusions regarding the shear rate can be drawn non-invasively. Moreover, the in vitro manometry performed here is unique, never having been performed previously, to the best of our knowledge. The analysis resembles the classical four sensor-based manometry used in clinical studies, with the added advantage of being non-invasive, since the pressure transducers are embedded in the model pharynx wall.

The current study is the first to involve clinical equivalent bolus visualisation and manometry being performed non-invasively. The device itself represents a valuable tool for the manufacturers of food thickeners to test novel formulations, as mentioned in the White Paper on fluid thickening [8].

## Conclusion

We show that, for thickened fluids, the velocity profiles for boluses with consistencies that range from honey like to pudding like give shear rates that range from 13 ± 6 s^−1^ to 209 ± 46 s^−1^. Thus, fluid characterisation at several shear rates other than just at 50 s^−1^ is warranted. Moreover, non-invasive in vitro manometry performed with the in vitro swallowing device, with the focus on bolus volume, consistency, and modified UES area, gives results similar to those seen in some clinical studies. Therefore, the device could be used as a pre-clinical examination tool, to improve our understanding of the linkages between bolus rheology and the biomechanics of swallowing.

## References

[1] Popa Nita S, Murith M, Chisholm H, Engmann J (2013). Matching the rheological properties of videofluoroscopic contrast agents and thickened liquid prescriptions. Dysphagia.

[2] Clavé P, De Kraa M, Arreola V, Girvent M, Farre R, Palomera E, Serra-Prat M (2006). The effect of bolus viscosity on swallowing function in neurogenic dysphagia. Aliment Pharmacol Ther.

[3] Tashiro A, Hasegawa A, Kohyama K, Kumagai H, Kumagai H (2010). Relationship between the rheological properties of thickener solutions and their velocity through the pharynx as measured by the ultrasonic pulse Doppler method. Biosci Biotechnol Biochem.

[4] Steele C, Alsanei W, Ayanikalath S, Barbon CA, Chen J, Cichero JY, Coutts K, Dantas R, Duivestein J, Giosa L, Hanson B, Lam P, Lecko C, Leigh C, Nagy A, Namasivayam A, Nascimento W, Odendaal I, Smith C, Wang H (2015). The influence of food texture and liquid consistency modification on swallowing physiology and function: a systematic review. Dysphagia.

[5] Burbidge AS, Cichero JAY, Engmann J, Steele CM (2016). A day in the life of the fluid bolus: An introduction to fluid mechanics of the oropharyngeal phase of swallowing with particular focus on dysphagia. Appl Rheol.

[6] Hasegawa A, Otoguro A, Kumagai H, Nakazawa F (2005). Velocity of swallowed gel food in the pharynx by ultrasonic method. J Jpn Soc Food Sci Technol.

[7] Zhu J, Mizunuma H, Michiwaki Y (2014). Determination of characteristic shear rate of a liquid bolus through the pharynx during swallowing. J Texture Stud.

[8] Newman R, Vilardell N, Clavé P, Speyer R (2016). Effect of bolus viscosity on the safety and efficacy of swallowing and the kinematics of the swallow response in patients with oropharyngeal dysphagia: White Paper by the European Society for Swallowing Disorders (ESSD). Dysphagia.

[9] Waqas MQ, Wiklund J, Altskar A, Ekberg O, Stading M (2017). Shear and extensional rheology of commercial thickeners used for dysphagia management. J Texture Stud.

[10] Moret-Tatay Rodríguez-García, Martí-Bonmatí J, Hernando I, Hernández MJ (2015). Commercial thickeners used by patients with dysphagia: rheological and structural behaviour in different food matrices. Food Hydrocoll.

[11] Leonard RJ, White C, McKenzie S, Belafsky PC (2014). Effects of bolus rheology on aspiration in patients with dysphagia. J Acad Nutr Diet.

[12] Meng Y, Rao MA, Datta AK (2005). Computer simulation of the pharyngeal bolus transport of Newtonian and non-Newtonian fluids. Food Bioprod Process.

[13] Salinas-Vázquez M, Vicente W, Brito-de la Fuente E, Gallegos C, Márquez J, Ascanio G (2014). Early numerical studies on the peristaltic flow through the pharynx. J Texture Stud.

[14] Wiklund J, Shahram I, Stading M (2007). Methodology for in-line rheology by ultrasound Doppler velocity profiling and pressure difference techniques. Chem Eng Sci.

[15] Omari TI, Wiklendt L, Dinning P, Costa M, Rommel N, Cock C (2014). Upper esophageal sphincter mechanical states analysis: a novel methodology to describe UES relaxation and opening. Front Syst Neurosci.

[16] Al-Toubi AK, Doeltgen SH, Daniels SK, Corey DM, Huckabee M-L (2015). Pharyngeal pressure differences between four types of swallowing in healthy participants. Physiol Behav.

[17] Cisonni J, Lucey AD, Walsh JH, King AJC, Elliott NSJ, Sampson DD, Eastwood PR, Hillman DR (2013). Effect of the velopharynx on intraluminal pressures in reconstructed pharynges derived from individuals with and without sleep apnea. J Biomech.

[18] Butler SG, Stuart A, Castell D, Russell GB, Koch K, Kemp S (2009). Effects of age, gender, bolus condition, viscosity, and volume on pharyngeal and upper esophageal sphincter pressure and temporal measurements during swallowing. J Speech Lang Hear Res.

[19] Ergun GA, Kahrilas PJ, Lin S, Logemann JA, Harig JM (1993). Shape, volume, and content of the deglutitive pharyngeal chamber imaged by ultrafast computerized tomography. Gastroenterology.

[20] Yue L, Guang-qing X, Fan Y, Tuo L, Li-sheng J, Feng L (2017). The effect of bolus consistency on swallowing function measured by high-resolution manometry in healthy volunteers. Laryngoscope.

[21] Chen FJ, Dirven S, Xu WL, Bronlund J, Li XN, Pullan A (2012). Review of the swallowing system and process for a biologically mimicking swallowing robot. Mechatronics.

[22] Morinière S, Hammoudi K, Marmouset F, Bakhos D, Beutter P, Patat F (2013). Ultrasound analysis of the upper esophageal sphincter during swallowing in the healthy subject. Eur Ann Otorhinolaryngol Head Neck Dis.

[23] Dirven S, Xu W, Cheng LK, Allen J (2015). Biomimetic investigation of intrabolus pressure signatures by a peristaltic swallowing robot. IEEE Trans Instrum Meas.

[24] Nystrom M, Qazi WM, Bülow M, Ekberg O, Stading M (2015). Effects of rheological factors on perceived ease of swallowing. Applied rheology.

[25] Force NDDT, Association AD. *National Dysphagia Diet: Standardization for Optimal Care*. 2002: American Dietetic Association.

[26] Stading M, Waqas MQ, Holmberg F, Kotze R, Kotze R, Ekberg O (2018). A device that models human swallowing. J dysphagia..

[27] Wiklund J, Johansson M, Shaik J, Fischer P, Windhab E, Stading M, Hermansson A-M. *In*-*line ultrasound based rheometry of industrial and model suspensions flowing through pipes.* Paper presented at the trans. third international symposium on ultrasonic Doppler methods for fluid engineering, EFPL, Lausanne, Switzerland. 2002.

[28] Wiklund J, Stading M, Trägårdh C (2010). Monitoring liquid displacement of model and industrial fluids in pipes by in-line ultrasonic rheometry. J Food Eng.

[29] Kotzé R, Wiklund J, Haldenwang R, Fester V (2011). Measurement and analysis of flow behaviour in complex geometries using the Ultrasonic Velocity Profiling (UVP) technique. Flow Meas Instrum.

[30] Hasegawa A, Nakazawa F, Kumagai H (2008). Velocity of swallowed food for dysphagic patients through the pharynx by ultrasonic method. Nippon Shokuhin Kagaku Kogaku Kaishi.

[31] Rofes L, Arreola V, Mukherjee R, Swanson J, Clavé P (2014). The effects of a xanthan gum-based thickener on the swallowing function of patients with dysphagia. Aliment Pharmacol Ther.

[32] Olthoff A, Zhang S, Schweizer R, Frahm J (2014). On the physiology of normal swallowing as revealed by magnetic resonance imaging in real time. Gastroenterol Res Prac.

[33] Nascimento WV, Cassiani RA, Santos CM, Dantas RO (2015). Effect of bolus volume and consistency on swallowing events duration in healthy subjects. J Neurogastroenterol Motil.

[34] Lin T, Xu G, Dou Z, Lan Y, Yu F, Jiang L (2014). Effect of bolus volume on pharyngeal swallowing assessed by high-resolution manometry. Physiol Behav.

[35] Dodds W, Man K, Cook I, Kahrilas P, Stewart E, Kern M (1988). Influence of bolus volume on swallow-induced hyoid movement in normal subjects. Am J Roentgenol.

[36] Hoffman MR, Ciucci MR, Mielens JD, Jiang JJ, McCulloch TM (2010). Pharyngeal swallow adaptations to bolus volume measured with high resolution manometry. Laryngoscope.

[37] Bhatia SJ, Shah C (2013). How to perform and interpret upper esophageal sphincter manometry. J Neurogastroenterol Motil.

